# THE LONG REACH OF SOCIAL RELATIONS: GENDER AND THE SES-HEALTH LINK

**DOI:** 10.1093/geroni/igae098.1165

**Published:** 2024-12-31

**Authors:** Toni Antonucci, Simon Brauer, Joonyoung Cho, Urmimala Ghose, Kristine Ajrouch

**Affiliations:** University of Michigan, Ann Arbor, Michigan, United States; University of Michigan, Ann Arbor, Michigan, United States; University of Hawaii, Hawaii, United States; Humboldt University Berlin, Berlin, Berlin, Germany; University of Michigan, Ann Arbor, Michigan, United States

## Abstract

Accumulated evidence demonstrates a strong relationship between socioeconomic status (SES) and health. We use longitudinal data over a period of 25 years to examine whether education (an established indicator of SES) predicts baseline health (i.e., intercept at W1) and its temporal change (i.e., slope from W1 through W3) in middle-aged and older adults. We further explore the long reach of social relations by testing whether social relations, particularly of parents with children (at W1), mediate and/or moderate the education–health link. The data are drawn from a regionally representative sample of adults (aged 40–93) in the Detroit area, USA. All analyses are stratified by gender (W1 N=males: 330; females: 468). A series of structural equation models were performed. For females, those with higher education had better health in W1. Their health declined across time and at the same rate as those with lower-education. Further, relationship quality did not mediate or moderate these relationships. Among males, those with higher education had better health in W1. Notably, the health of men with lower-education who had a child with whom they confided and those with higher education had similar health at wave 1. Findings indicate that the health of lower-educated men in the presence of key social supports parallels the advantaged health status of men with higher levels of education earlier in life but diverges later in life. These findings suggest that relationships with children may be a protective factor for the health of men in the lower socioeconomic strata.

